# Characterization of Defects in Ion Transport and Tissue Development in Cystic Fibrosis Transmembrane Conductance Regulator (CFTR)-Knockout Rats

**DOI:** 10.1371/journal.pone.0091253

**Published:** 2014-03-07

**Authors:** Katherine L. Tuggle, Susan E. Birket, Xiaoxia Cui, Jeong Hong, Joe Warren, Lara Reid, Andre Chambers, Diana Ji, Kevin Gamber, Kengyeh K. Chu, Guillermo Tearney, Li Ping Tang, James A. Fortenberry, Ming Du, Joan M. Cadillac, David M. Bedwell, Steven M. Rowe, Eric J. Sorscher, Michelle V. Fanucchi

**Affiliations:** 1 Gregory Fleming James Cystic Fibrosis Research Center, University of Alabama at Birmingham, Birmingham, Alabama, United States of America; 2 Department of Environmental Health Sciences, School of Public Health, University of Alabama at Birmingham, Birmingham, Alabama, United States of America; 3 Department of Medicine, University of Alabama at Birmingham, Birmingham, Alabama, United States of America; 4 SAGE Labs, Inc., St. Louis, Missouri, United States of America; 5 Department of Cell, Developmental, and Integrative Biology, University of Alabama at Birmingham, Birmingham, Alabama, United States of America; 6 Wellman Center for Photomedicine, Harvard Medical School, Massachusetts General Hospital, Boston, Massachusetts, United States of America; 7 Department of Dermatology, Massachusetts General Hospital, Boston, Massachusetts, United States of America; 8 Department of Pathology, Massachusetts General Hospital, Boston, Massachusetts, United States of America; 9 Animal Resources Program, Office of the Vice President for Research, University of Alabama at Birmingham, Birmingham, Alabama, United States of America; 10 Department of Microbiology, University of Alabama at Birmingham, Birmingham, Alabama, United States of America; The Ohio State University, United States of America

## Abstract

Animal models for cystic fibrosis (CF) have contributed significantly to our understanding of disease pathogenesis. Here we describe development and characterization of the first cystic fibrosis rat, in which the cystic fibrosis transmembrane conductance regulator gene (CFTR) was knocked out using a pair of zinc finger endonucleases (ZFN). The disrupted *Cftr* gene carries a 16 base pair deletion in exon 3, resulting in loss of CFTR protein expression. Breeding of heterozygous (CFTR^+/−^) rats resulted in Mendelian distribution of wild-type, heterozygous, and homozygous (CFTR^−/−^) pups. Nasal potential difference and transepithelial short circuit current measurements established a robust CF bioelectric phenotype, similar in many respects to that seen in CF patients. Young CFTR^−/−^ rats exhibited histological abnormalities in the ileum and increased intracellular mucus in the proximal nasal septa. By six weeks of age, CFTR^−/−^ males lacked the vas deferens bilaterally. Airway surface liquid and periciliary liquid depth were reduced, and submucosal gland size was abnormal in CFTR^−/−^ animals. Use of ZFN based gene disruption successfully generated a CF animal model that recapitulates many aspects of human disease, and may be useful for modeling other CF genotypes, including CFTR processing defects, premature truncation alleles, and channel gating abnormalities.

## Introduction

Cystic fibrosis (CF) is the most common lethal recessive genetic disorder among individuals of European descent, affecting 1 in every 2,500–3,500 newborns each year [1]. The disease is characterized by multi-system pathology, including respiratory complications, intestinal obstruction, exocrine pancreatic disease, hepatoductal blockage, and absence of the vas deferens [2]. The predominant cause of morbidity and mortality in CF results from chronic pulmonary infection and inflammation. CF is caused by mutations in the cystic fibrosis transmembrane conductance regulator (CFTR) gene, encoding an anion channel expressed in epithelial and other tissues.

A variety of CF mice have been generated since the discovery of *Cftr* in 1989 [3], [4]. The mouse models, while presenting with CF-related intestinal disease, fail to recapitulate many other manifestations observed in patients. Nonetheless, CF mice have provided a valuable tool for testing pharmaceutical and other interventions, and investigating contributors to pathogenesis, including CF modifier genes [5]. Porcine [6] and ferret [7] CF models exhibit a respiratory phenotype closely resembling that observed in humans, although prolonged gestational period, time to sexual maturation, expense, and specialized care requirements have significantly limited their widespread use.

The development of a CF rat (*Rattus norvegicus)* would provide a number of advantages in comparison with available animal models of CF. First, the rat has a very short gestational period (21–23 days) and time to sexual maturity (8 weeks), allowing rapid colony propagation, breeding studies, and characterization of animals as they mature shortly after birth. Second, there is considerable interest regarding airway glandular function as a mediator of CF respiratory failure [8]. Airway submucosal glands are believed to underlie considerable pathology observed in human CF lungs. Rats are an attractive model in this context because, unlike mice but similar to humans, rats develop extensive submucosal glands throughout the trachea to the level of bronchi [9]. Third, relative to mice, rats are considerably larger in size, even during the early postnatal period, allowing for larger tissue samples to be collected from animals and ease performing surgical procedures [10]. Rats are also a traditional species for pharmacology and toxicology research due to their well-defined pharmacokinetic and biodistribution profiles [11], [12]. Previous CF studies have sometimes required transgenic mice for efficacy, with safety studies conducted in rat; however, a CF rat model would facilitate pharmaceutical efficacy and safety studies of potential therapeutic molecules in the same species. Finally, because rats have been well studied in the laboratory for years, there is a large body of literature regarding normal physiology and a vast array of laboratory tools and reagents (i.e. antibodies, siRNA, other genomic probes) readily available for the study of chaperone, binding partner, and other protein based analyses relevant to disease mechanism that would be very difficult to obtain for ferret or pig.

Recent advances in gene manipulation techniques have provided a number of opportunities for developing genetically modified animals other than mouse. Zinc-finger endonuclease (ZFN) technology, for example, allows targeting of user-defined site-specific mutations that generate knockout animals with high efficiency and over a shorter time line than embryonic stem-cell targeting used in many species other than mouse [13], [14]. Here, we describe the generation of a CFTR^−/−^ rat by pronuclear microinjection of ZFNs and its characterization. The disease phenotypes observed in young (21–44 days postnatal) CFTR^−/−^ animals, which closely resemble human manifestations, suggest the rat model will be useful for studies of CF pulmonary pathogenesis and drug development.

## Materials and Methods

### Ethics Statement

This study was carried out in compliance with the Guide for the Care and Use of Laboratory Animals of the National Institutes of Health. Protocols were approved by the SAGE Labs, Inc. or University of Alabama at Birmingham (UAB) Institutional Animal Care and Use Committee (IACUC; SAGE Approval Number 001.02, UAB Approval Number 09479). All surgeries were performed under sodium pentobarbital or ketamine/xylazine/acepromazine anesthesia with all efforts made to minimize animal suffering.

### Generation of the Model

#### ZFN mRNA preparation

*Cftr* specific ZFNs were obtained from the CompoZr product line (Sigma, St. Louis, MO). mRNA was prepared from each construct, linearized with XhoI and modified using MessageMax and Poly(A) polymerase tailing kits (Epicentre Biotechnology). Samples were purified, quantified, and transfected at a 1∶1 ratio into rat C6 cells for activity validation.

#### Animal husbandry

Derivation and breeding of animals was conducted at SAGE Labs (microinjection and founder identification/breeding) operated under approved animal protocols overseen by the SAGE IACUC. Sprague Dawley rats (Ntac:SD) from Taconic Farms (Hudson, New York) were used for microinjection. Animals were bred with housing in standard cages maintained on a 12 h light/dark cycle with *ad libitum* access to food and water. Routine health monitoring of the colony was performed at IDEXX (Columbia, MO) and indicated no evidence of infection with known serious pathogens.

#### Microinjection

Four to five week-old female donors were injected with 20 units of pregnant mare serum gonadotropin (PMS) followed by injection of 50 units hCG after an additional 48 h, and immediately mated with stud males. Fertilized eggs were harvested a day later. *Cftr* ZFN mRNA was microinjected at 10 ng/ml into the pronucleus of fertilized eggs. Following microinjection, 25–30 eggs were transferred into each pseudopregnant female, leading to birth of the founder generation.

#### Founder identification and breeding

Tail or toe biopsies were used for genomic DNA extraction and analysis as described previously [15]. Primers flanking the target site were forward 5′- AATATCTGGGTGGGCAGTTG and reverse 5′-TTGTTTGCAAGATTGCCCTT. Primers used to detect larger deletions were LD forward 5′-TACGCAATGCCAAGAAGTCA; LD reverse 5′-GAGGATGTTGGGAAGCTTTG. A founder was selected and bred with wild-type to obtain heterozygous animals, and sibling mating of heterozygotes resulted in homozygous SD- *CFTR^tm1sage^* rats (termed CFTR^−/−^).

### Characterization of the Model

#### Animals

All animal experiments at UAB were conducted in accordance with UAB IACUC approved protocols. Male and female Sprague-Dawley CFTR^+/−^ rats were paired and housed in HEPA filtered ventilated cages with a 12 hour light/dark cycle and provided sterilized food and water *ad libitum*. Litters remained with lactating dams until weaning. Heterozygous rats were separated from wild-type and CFTR^−/−^ littermates. Heterozygous animals exhibited growth and survival rates similar to wild-type, did not develop intestinal obstruction, and presented with normal dentition and complete male reproductive organs. In preliminary studies, CFTR heterozygotes also exhibited similar bioelectric and other characteristics to wild-type. In order to preclude any subtle confounding variables, CFTR+/− carriers were excluded from data analysis shown in this manuscript. Wild-type and CFTR^−/−^ animals were provided water and standard rodent chow (pellet and ground) with a supplemental diet of DietGel 76A (Clear H_2_O). A subset of wild-type and CFTR^−/−^ pups were also provided 1× GoLytely (Braintree Laboratories, Inc) at weaning as a means to reduce gastrointestinal complications.

#### PCR genotyping

DNA analysis of litters was performed by extracting tail snip genomic DNA prepared in 500 µL lysis buffer (200 mM NaCl, 100 mM Tris-HCl, 5 mM EDTA, 0.25% Tween-20) with 1 mg Proteinase K (Sigma P6556) overnight at room temperature, followed by 60°C incubation to complete tissue lysis. DNA was obtained by standard techniques with 2-propanol, 70% ethanol, and DNA stored TE buffer (10 mM Tris-HCl, 1 mM EDTA) at −20°C. For PCR, 1 µL of DNA was mixed with 8 µL H_2_0, 10 µL JumpStart Taq ReadyMix (Sigma P2893), and 0.5 µL each primer (forward 5′-GCAGCTCACTGGTCGATCTT, reverse 5′-GACACTATATTCACAAGGGAGAG). PCR conditions were 95°C for 5 minutes, followed by 35 cycles of 95°C for 30 seconds, 60°C for 30 seconds, and 68°C for 40 seconds, with a fixed cycle at 68°C for 5 minutes. This PCR resulted in amplification of a 172-bp DNA fragment for the wild-type *Cftr* allele and a 156-bp DNA fragment (16-bp deleted) for mutant *Cftr*. PCR products were resolved on a 3.5% agarose gel.

#### Western blotting

Lung tissue was homogenized in TBS on ice followed by lysis in RIPA buffer (ThermoScientific, Rockford, Il) with Halt protease inhibitor cocktail (ThermoScientific). Protein was quantitated using the BCA assay (ThermoScientific), samples mixed with 4× sample buffer, and incubated at 37°C for 10 minutes. Equal amounts of protein (20 µg) were loaded into each lane, resolved by SDS-PAGE, and blotted onto PVDF membranes. Wild-type Sprague-Dawley rat lung extract (Sc-2396, Santa Cruz Biotechnology, Inc, Dallas, Tx) was used as an additional positive control for CFTR detection. Blocking was with 1% rabbit serum followed by incubation with goat anti-CFTR primary antibody (1∶200 Sc-8909) overnight at 4°C, and subsequent rabbit anti-goat HRP conjugated secondary antibody (1∶5000 Sc-2768) for 1 hour at room temperature. Labeled proteins were detected using SuperSignal West Femto ECL kit (ThermoScientific).

#### Histology

Left lung lobes were cannulated and inflation fixed with 1% paraformaldehyde at 30 cm pressure for 30–60 minutes, followed by storage in 10% buffered formalin. Nasal tissue was collected, mandible removed, and nasal passages flushed retrograde with 10% buffered formalin followed by immersion in formalin until processed [16]. Nasal samples were decalcified for 4–5 days in Immunocal (Decal Chemical Corporation, Tallman, NY) solution, and rinsed thoroughly. The nasal cavity was sectioned into four regions at specific anatomic sites as described previously [17], [18]. All other tissues were harvested, immersion fixed in 10% buffered formalin or 70% ethanol/formalin and stored at 4°C until processed. Tissues were embedded in paraffin, sectioned, and stained with hematoxylin and eosin (H&E) for evaluation by a board certified veterinary pathologist. Nasal, tracheal, lung, and intestinal tissues were stained with alcian blue periodic acid Schiff (AB-PAS) for identification of mucosubstances. Sections were imaged on an Olympus BX-41 microscope with a digital Q-color 5 camera (Olympus) using Q Capture imaging software (Q Imaging, Surrey, Canada) [19].

#### Quantitation of intracellular mucus in the nasal septa

The amount of stored mucosubstance in upper respiratory epithelium was estimated by quantifying the area of AB-PAS positive tissue per unit basement membrane. Septa from proximal nasal samples were evaluated at 20×, providing 4–6 fields (entire length of septa) per animal. Images were imported into ImageJ (NIH) and colors separated using the RGB stack feature. Using a green channel, areas of interest were outlined using the polygon tool and maximum threshold set for each image to include all stained intracellular mucus.

#### Morphometric analysis of tracheal tissue

Tracheas were imaged at 4× and 20× magnification followed by analysis with ImageJ (NIH) using protocols modified from Meyerholz *et al.* [20]. For each animal, three tracheal sections were studied (with a minimum distance of 15 µm between sections) using the following parameters. Lumenal circumference was determined by measuring the apical surface of the epithelium. Cartilage area was quantified by outlining cartilaginous tissue in the tracheal ring, measuring the area, and summing area for all cartilage in each image. Maximal cartilage thickness was evaluated by determining the perpendicular distance between outer boundaries of cartilage rings, with epithelial thickness represented by the distance between the basement membrane (on the serosal surface) and the apical membrane lumenally. Submucosal gland area was determined by circling and measuring both serous and mucus components of the gland structures. Submucosal gland intracellular mucus was assessed by outlining the entire submucosal gland area (same protocol as for quantitation of intracellular mucus in the nasal septa), in order to define the extent of intracellular mucosubstance. The percent of intracellular mucus was determined as the ratio of intracellular mucus/total submucosal gland area.

### Bioelectric Measurements

#### Nasal potential difference

Rats were anesthetized with ketamine (200 mg/kg), acepromazine (0.6 mg/kg), and xylazine (30 mg/kg) by intraperitoneal injection. Potential difference was measured using AgCl electrode and electronic data capture (AD Instruments) as previously described for mouse and human [21], [22]. Nasal cavities were perfused sequentially with 1) Ringer solution containing 140 mM NaCl, 5 mM KCl, 1 mM MgCl_2_, 2 mM CaCl_2_, 10 mM HEPES, and 100 µM amiloride (pH 7.3); 2) Cl^–^free Ringer solution (6 mM Cl^−^, pH 7.3) with amiloride; and 3) Cl^–^free Ringer solution, amiloride, and forskolin (20 µM). Each condition was studied for 5 to 10 minutes until a stable signal was achieved. Traces were interpreted in a blinded fashion.

#### Tracheal short-circuit current (I_SC_)

Tracheas were excised, sectioned into 3–4 segments, and opened longitudinally along the dorsal surface. Segments were mounted as flat sheets in modified Ussing chambers (area ∼0.031 cm^2^) maintained at 37°C and bubbled vigorously with 95% O_2_∶5% CO_2_.

I_SC_ measurements were performed under voltage clamp conductions using MC8 equipment and P2300 Ussing chambers (Physiologic Instruments, San Diego, CA). Tissue segments were equilibrated for 10 minutes in regular Ringer solution that contained (in mM) 120 NaCl, 25 NaHCO_3_, 3.33 KH_2_PO_4_, 0.83 K_2_HPO_4_, 1.2 CaCl_2_, 1.2 MgCl_2_, and 10 mannitol to establish a baseline and then tested by one of the following experimental protocols:

1. Administration of amiloride (100 µM) to inhibit the epithelial sodium channel (ENaC), followed by sequential addition of forskolin (10 µM) to activate cAMP-dependent CFTR current, ATP (10 µM) to activate Ca^2+^- activated chloride channel (CaCC) transport, and bumetanide (100 µM) to block transepithelial Cl^−^ transport.
2. Administration of CFTR_Inh_-172 (10 µM) to block constitutively active CFTR dependent chloride current, followed by sequential addition of amiloride, ATP, and bumetanide as above.

Changes in I_SC_ attributable to ion transport agonists and inhibitors were calculated following achievement of a stable plateau for several minutes. ATP-sensitive I_SC_ was measured as the highest current value for each sample [21].

#### Ileal I_SC_ measurements

Tissue segments approximately 8 mm in length sectioned 5 cm above the cecum were stripped of the serosa (visceral peritoneum) and longitudinal/circular muscle layers of the intestinal wall, opened longitudinally along the mesenteric border, and incubated in TTX (Tetrodotoxin, 3.3×10^−4^ µM in PBS) for 10 min to block action potential dependent sodium channels. Segments were mounted as flat sheets onto sliders (area ∼0.09 cm^2^) and I_SC_ measured under voltage clamp conditions using MC8 equipment and P2300 Ussing chambers (Physiologic Instruments, San Diego, CA) as previously described [23]. Bath solutions were gently stirred and gassed with 95% O_2_∶5% CO_2_.

Regular Ringer solution was utilized for monitoring I_SC_ as above. Low Cl^−^ Ringer contained (in mM) 1.2 NaCl, 25 NaHCO_3_, 3.33 KH_2_PO_4_, 0.83 K_2_HPO_4_, 1.2 CaCl_2_, 1.2 MgCl_2_, 141 Na gluconate, and 10.8 mannitol. Sliders with mounted ileum were equilibrated for 10 minutes in Ringer solution followed by 10 minutes of recording; mucosal side chambers were changed to 1∶1 regular Ringer:low Cl^−^ Ringer. Indomethacin (10 µm) was added to both chambers to block ion transport associated with phospholipase C or A2 activity induced by seromuscular stripping. After 30 minutes of incubation, forskolin (10 µm) and IBMX (3-Isobutyl-1-methylxanthine, 100 µm) were added to both chambers, followed by glybenclamide (200 µM) to block forskolin-activated CFTR short-circuit current.

In all experiments, fully stimulated current was obtained within 3 minutes of forskolin addition. Pulse during voltage clamp measurements was imposed every second after forskolin/IBMX stimulation, and every 20 seconds following other drug treatments.

### Micro Optical Coherence Tomography (µOCT)

#### Tissue preparation

Tracheal tissue was excised, immediately placed on Gelfoam soaked in DMEM/F12 1: 1 media, and incubated under physiologic conditions (37°C, 5% CO_2_, and 100% humidity) using an environmentally controlled chamber (Pathology Devices, Westminster, MD). Tracheas were equilibrated for 30 minutes before analysis.

#### Image acquisition

Measurements of functional microanatomic parameters in tracheal tissue, including measurement of (i) airway surface liquid (ASL) depth, the aqueous layer lining the airway epithelium, (ii) periciliary liquid (PCL) depth, the thin aqueous gel surrounding the cilia, (iii) ciliary beat frequency (CBF), and (iv) velocity of mucociliary transport (MCT) were performed using Micro-Optical Coherence Tomography (µOCT), a high-speed, high-resolution microscopic reflectance imaging modality [24]. µOCT methods for investigation of airway epithelia and quantitative image analysis have been previously described [24], [25]. In brief, the µOCT instrument provides cross-sectional images of epithelium with a transverse and axial resolutions of approximately 2 µm and 1 µm, respectively. This resolution is sufficient to directly visualize and quantify parameters including ASL depth, PCL depth, CBF, and MCT rate without using exogenous dyes or particles. Acquisition speed is 20,480 Hz line rate, resulting in 40 frames per second at 512 lines per frame.

#### Image analysis

Quantitative analysis of ASL and PCL depths were characterized directly by geometric measurement of the respective layers, and images over several frames captured the length of fully extended cilia. CBF was investigated by Fourier analysis of the reflectance due to beating cilia. MCT rate was determined using time elapsed and distance traveled of native particulates in the mucus over multiple frames. Images were acquired at randomly chosen locations on the mucosal surface with the optical beam scanned along the longitudinal axis of the trachea.

#### Bronchoalveolar lavage fluid cell differentials

Tracheas were cannulated and lungs lavaged with 2.0 mL PBS (7.4 pH). Cells were collected by instilling 2.0 mL phosphate buffered saline (pH 7.2) and the lavage centrifuged onto slides (Cytospin). Slides were stained using Hema 3 stain kit (Fisher Scientific, Kalamazoo, MI) [26]. A minimum of 300 cells were counted per animal.

#### Complete Blood Counts (CBC) and serum chemistry

Blood (200–500 µL) was collected from the brachial artery into EDTA tubes and processed for CBC by ANATECH Diagnostics (Smyrna, GA). For serum chemistry, whole blood (300–500 µL) was processed in serum separator tubes and allowed to clot at room temperature for 1 hour. Supernatant was obtained by centrifugation at 10,000×*g* for 10 minutes, with serum placed in a fresh tube and frozen at −80°C. Samples were thawed and analyzed on an Abaxis VetScan using a Comprehensive Diagnostic Profile rotor (500–1038, Union City, CA).

#### Statistical analysis

Data are expressed as mean ± SEM or as individual data points. Statistical significance was determined using unpaired, two-tailed Student’s t-test or one-way ANOVA using Prism (GraphPad, LaJolla, CA). For survival analysis, Kaplan-Meier survival curves were plotted and statistical significance determined using the logrank test for trend. P values ≤0.05 were considered significant.

## Results

### Microinjection and Founder Identification

**Figure 1:**
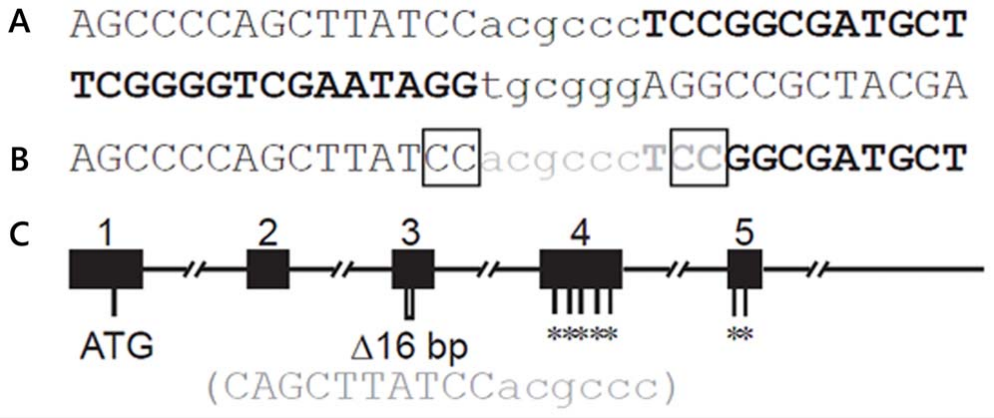
Targeting in exon 3 of *Cftr*. (A) ZFN recognition site sequence. The two ZFN binding sites are in bold uppercase. Cleavage site is in lower case. (B) Nine base pair deletion recovered in multiple founders. The deleted sequence is shown in gray. Microhomology that may have favored this deletion is marked in boxes. (C)Schematic of the gene structure of the first 5 exons of rat *Cftr*. Exons are shown by filled rectangles with exon number above. ATG, position of the translational start codon; Δ16 bp marks the position of 16 bp deletion, with nucleotide sequence below). *indicates premature stop mutations introduced by the 16 bp deletion in exon 3.

A pair of ZFNs was confirmed to cleave the target site within exon 3 of *Cftr*, shown in Figure 1A. Following microinjection and embryo transfer, 44 pups were born to six recipients, 18 of which carried at least one mutant allele. The mutations ranged from 9 bp to hundreds of base pairs in length. Seven founders carried the same 9 bp deletion (Figure 1B), presumably due to microhomology within the target site, as discussed previously [15]. One founder was mosaic, harboring wild type *Cftr* allele, an allele with a 16 bp deletion and an allele with a 479 bp deletion. Larger deletions were observed to span the junction of exon 3/intron 3. Heterozygous rats with the 16 bp deletion (a frameshift in the reading frame leading to premature termination in exon 4) (Figure 1C), were used to establish a breeding colony.

### Litter Demographics

**Figure 2:**
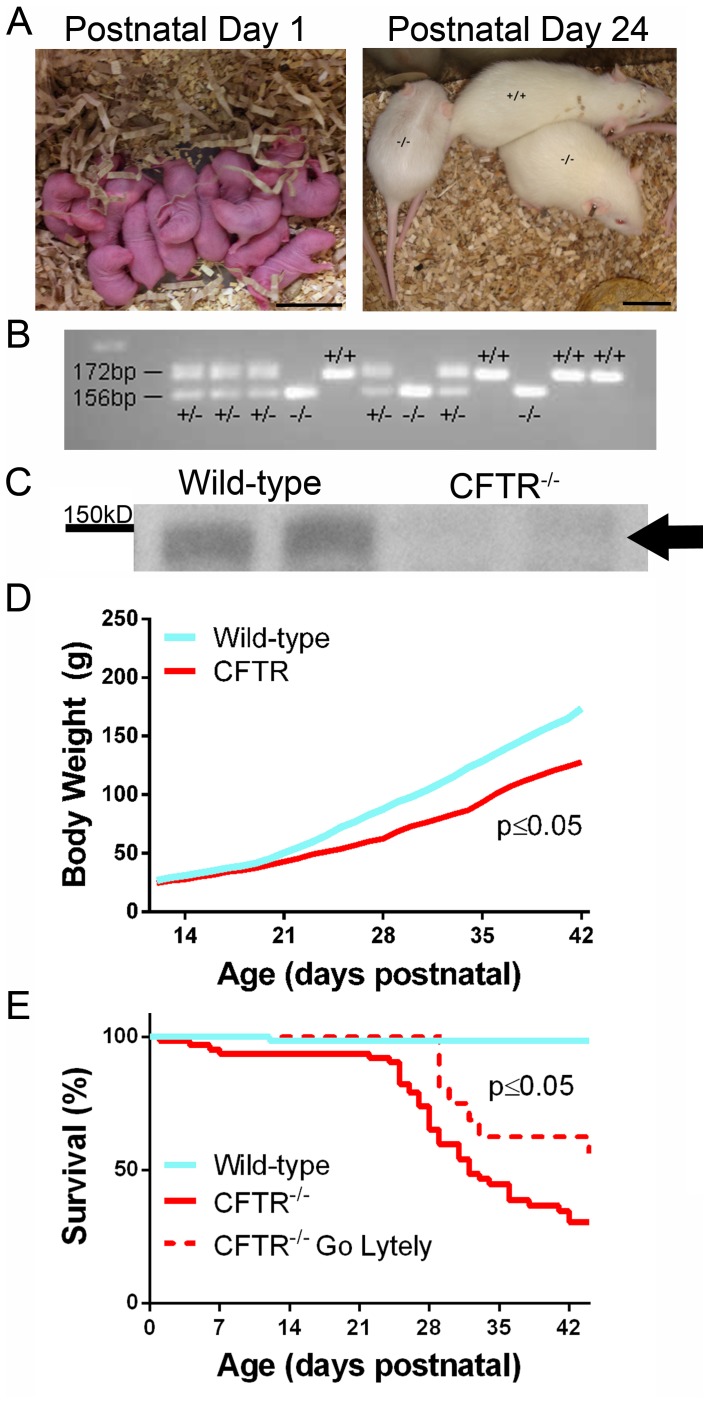
Generation of CFTR^−/−^ rats. (A) Animals at days 1 and 24 postnatal. (B) Results of PCR genotyping from a representative litter. (C) Western blot indicating expression of CFTR in wild-type rats and absence of CFTR protein from lungs of CFTR^−/−^ animals. Arrow - CFTR. (D) Body weight values (mean ± SD) from wild-type and CFTR^−/−^ rats from 12 to 44 days postnatal. (E) Survival curve for CFTR^−/−^ rats from postnatal day 1 to 44 (p<0.05 for all groups, n = 12–67 animals/group).

Four breeding pairs of CFTR^+/−^ rats gave rise to 332 pups from 30 litters with an average litter size of 11 pups. Litters were analyzed by PCR (Figure 2B) and found to have a genotype distribution of 27.1% wild-type, 49.4% heterozygous, and 23.5% CFTR^−/−^, a result similar to the expected 1∶2: 1 inheritance pattern. Western blot of whole lung homogenates from wild-type and CFTR^−/−^ animals confirmed that the 16 bp deletion resulted in loss of CFTR protein expression (Figure 2C and Figure S3). At birth, body weight and overall body size were similar between wild-type and CFTR^−/−^ animals; however, begining in the second week following birth, CFTR^−/−^ rats gained weight more slowly than wild-type littermates (Figure 2A, 2D). Survival of CFTR^−/−^ rats was similar to wild-type until weaning, but was greatly reduced by 6 weeks (30% vs 99%, p≤0.05; Figure 2E). Mortality was associated with weight loss secondary to gastrointestinal complications (including obstruction, see also below). Addition of GoLytely to water resulted in significantly improved survival rates of CFTR^−/−^ animals at 6 weeks of age (30% vs 64%, p≤0.05; Figure 2E).

### Ileal Tissue

**Figure 3:**
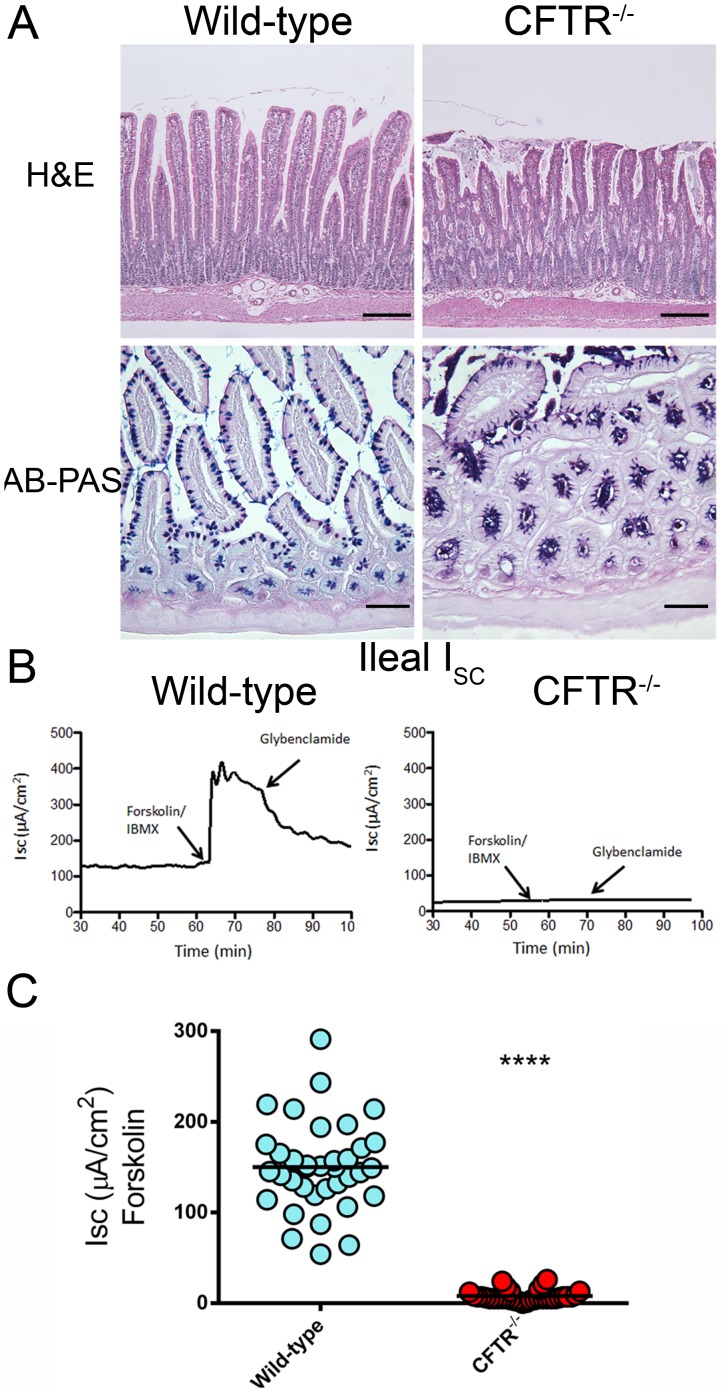
Histology and short-circuit current measurements from small intestines of wild-type and CFTR^−/−^ rats. H&E (bar = 200 µm) and AB-PAS (bar = 100 µm) stained sections of the small intestines from wild-type and CFTR^−/−^ rats. (n = 3–5 animals/group) (B) I_SC_ tracings from wild-type and CFTR^−/−^ rat ileum. (C) Summary of forskolin stimulated current measurements from ileal sections. (n = 5 animals/group) ****p≤0.0001.

CFTR^−/−^ rats developed intestinal obstruction after weaning and exhibited significant weight loss and decreased survival. Histological evaluation of small intestine demonstrated epithelial cell sloughing and crypts dilated with mucus, as well as a qualitative increase in bacterial load (Figure 3A). Ileal tissue was evaluated for CFTR short-circuit current phenotype by Ussing chamber measurements. Wild-type ileal tissue exhibited a strong forskolin-stimulated current (150±49 µA/cm^2^), which was absent in CF rats (8±6 µA/cm^2^, p<0.0001; Figures 3B, C).

### Stored Mucosubstances in Airway Epithelium of the Proximal Septa

**Figure 4:**
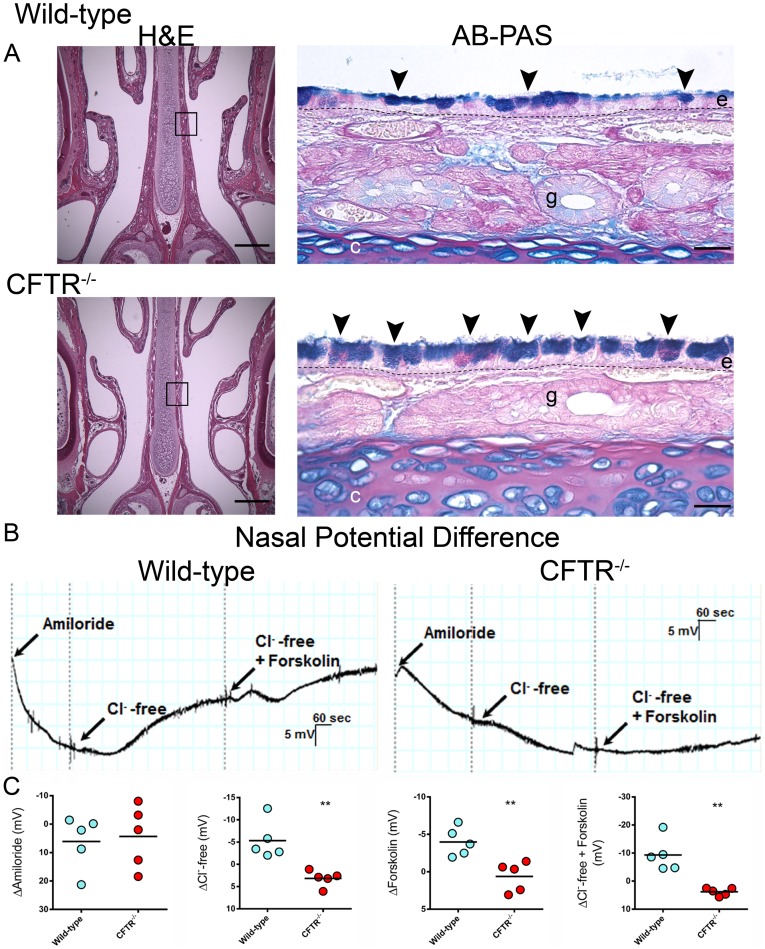
Proximal nasal histology and nasal potential difference measurements. (A) Low power magnification (4×) H&E stained sections from the proximal nasal passages bar = 500 µm. 20× images of ABPAS stained nasal septa from boxed areas bar = 25 µm. Arrowheads, cells swollen with intracellular mucus; e, respiratory epithelium; g, submucosal gland; dashed line (–), basement membrane (n = 4 animals/group) (B) NPD tracings from wild-type and CFTR^−/−^ rats. (C) Summary data from NPD measurements for Δamiloride, ΔCl^−^-free Ringers, Δforksolin, and ΔCl^−/−^free Ringers+forskolin. (n = 5 animals/group) **p≤0.01.

The respiratory epithelium of the nasal septum, immediately posterior to the upper incisors, was evaluated and labeled for intracellularly stored mucosubstance. Both wild-type and CFTR^−/−^ animals had normally developed epithelium consisting of a pseudostratified columnar epithelium comprised of both goblet and ciliated epithelial cells. CFTR^−/−^ animals had elevated levels of intracellular mucus that encompassed a significant portion of the airway epithelial cytosol (Figure 4A, Table 1), resulting in cells from CF animals appearing thicker and swollen (“stuffed”) compared to wild-type.

**Table 1:** Morphometric analysis of respiratory tissue from 3–6 week old wild-type and CFTR^−/−^ rats.

	Wild-type	CFTR^−/−^
**Nasal Septa**		
Intracellular mucus/basement membrane (µm^2^/µm)	3.5±0.9	7.5±0.9*
**Trachea**		
Lumen circumference (mm)	5.5±0.3	5.1±0.2
Cartilage area (mm^2^)	0.63±0.06	0.42±0.03**
Cartilage area/lumenal circumference (mm^2^/mm)	0.11±0.01	0.08±0.01*
Maximum cartilage thickness (mm)	0.20±0.01	0.17±0.01
Epithelial thickness (µm)	11.5±0.7	9.9±1.09
Submucosal gland area (mm^2^)	0.057±0.005	0.032±0.006*
Submucosal gland area/lumenal circumference (mm^2^/mm)	0.016±0.001	0.006±0.001*
Submucosal gland intracellular mucus (µm^2^)	7409±1481	2877±752*
% intracellular mucus	12±2	8.4±2

Values shown as mean ± SEM (n = 4–6 animals/group).

*p≤0.05, **p≤0.001 (Student’s t-test).

### Nasal Potential Difference Measurements

To characterize electrophysiology of the upper airway, transepithelial potential difference was monitored in response to a series of pharmacologic ion channel regulators. CFTR^−/−^ rats had no evidence of Cl^−^ dependent secretion upon stimulation of CFTR mediated Cl^−^ transport by Cl^–^free Ringer with forskolin (3.8±0.6 mV), whereas changes were robust in wild-type littermates (−9.3±2.7 mV, p≤0.01; Figure 4B, C). There were no differences between CF and non-CF following perfusion with amiloride. These measurements indicate a bioelectric phenotype consistent with absence of CFTR in the nasal airways, but without concomitant increase in amiloride sensitive voltage, the conventional ion transport pathway associated with sodium reabsorption.

### Morphometric Analysis of Tracheal Rings

**Figure 5:**
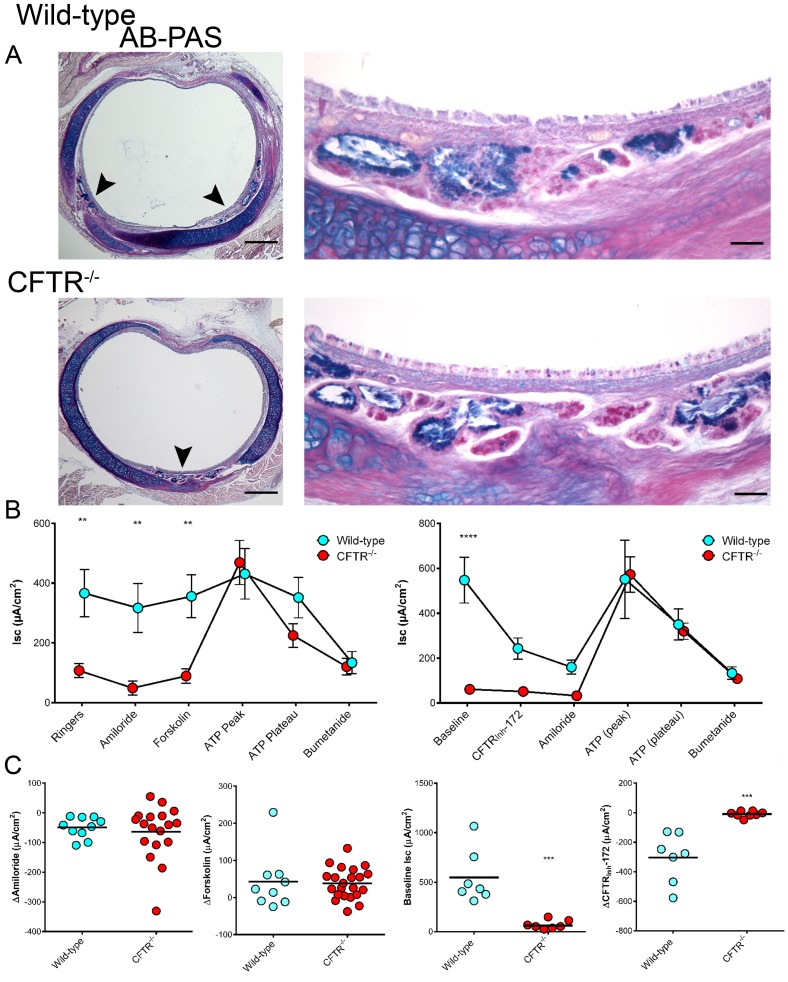
Tracheal histology and short circuit current measurements. (A) AB-PAS stained tracheal sections from 6 week old rats. Submucosal glandular tissue indicated by arrowheads. Low magnification bar = 500 µm, high magnification bar = 50 µm (B & C) Summary data from Ussing chamber short circuit current measurements. Panels on right depict a modified protocol designed to specifically detect baseline (constitutive) CFTR function. (n = 3–6 animals/group) **p≤0.01, ***p≤0.001, ****p≤0.0001.

Tracheal morphology in young CF animals appeared grossly similar between wild-type and CFTR^−/−^ genotypes (Figure 5); however, morphometric analysis revealed important differences attributable to absent CFTR (Table 1). While lumenal circumference was not significantly different between wild-type and CFTR^−/−^ rats at 3–6 weeks after birth, cartilage area was significantly diminished in CF animals. CF rats also exhibited reduced tracheal gland area (∼50% compared to wild-type) even after normalization for tracheal lumen circumference. Additionally, at all time points during the 3–6 week growth period, alcian blue positive (AB+) staining of submucosal glandular cells was decreased in CF versus wild-type rats, suggesting that glandular maturation in the CF animals was delayed in comparison to age-matched controls.

### Tracheal I_SC_ Measurements

Baseline I_SC_ of freshly excised trachea was significantly lower in CFTR^−/−^ animals compared to wild-type (Figure 5B), a finding predominantly attributable to CFTR_Inh_-172 sensitive current. To determine the contribution of Na^+^ transport to basal currents, amiloride was added apically (Figure 5B, left panel). The reduction in I_SC_ was similar for both wild-type and CFTR^−/−^ rats (49±11 µA/cm^2^ and 64±21 µA/cm^2^, respectively; Figure 5C) indicating comparable levels of sodium transport irrespective of CFTR expression (Figure 5B, left panel; a finding similar to the nasal ion transport phenotype, Figure 4). Subsequent addition of forskolin did not confer substantial I_SC_ activation in either group, suggesting CFTR may be constitutively active in wild-type sections, diminishing the additional activation otherwise expected from forskolin. In contrast, ATP stimulated current was much greater for CFTR^−/−^ animals (although maximal Cl^−^ currents for both genotypes were comparable and inhibited by addition of bumetanide), suggesting a compensatory increase of ATP-dependent Cl^−^ transport in the absence of functional CFTR, as also seen in humans [27]. To confirm that CFTR was constitutively active in wild-type animals, an alternative protocol was developed to test this interpretation (Figure 5B, right panel). Using the modified assay, wild-type rats demonstrated a baseline current of 547±102 µA, while current was far less (61±17 µA) in CFTR^−/−^ animals. Addition of CFTR_Inh_-172 reduced the current in wild-type by 304±63 µA, demonstrating high level basal CFTR activity, whereas CFTR_Inh_-172 had minimal effect on constitutive current in CFTR^−/−^ rats (9±7 µA, p≤0.001). ATP sensitive currents were strongly inhibited by the addition of bumetanide.

### Measurement of Functional Airway Microanatomy

**Figure 6:**
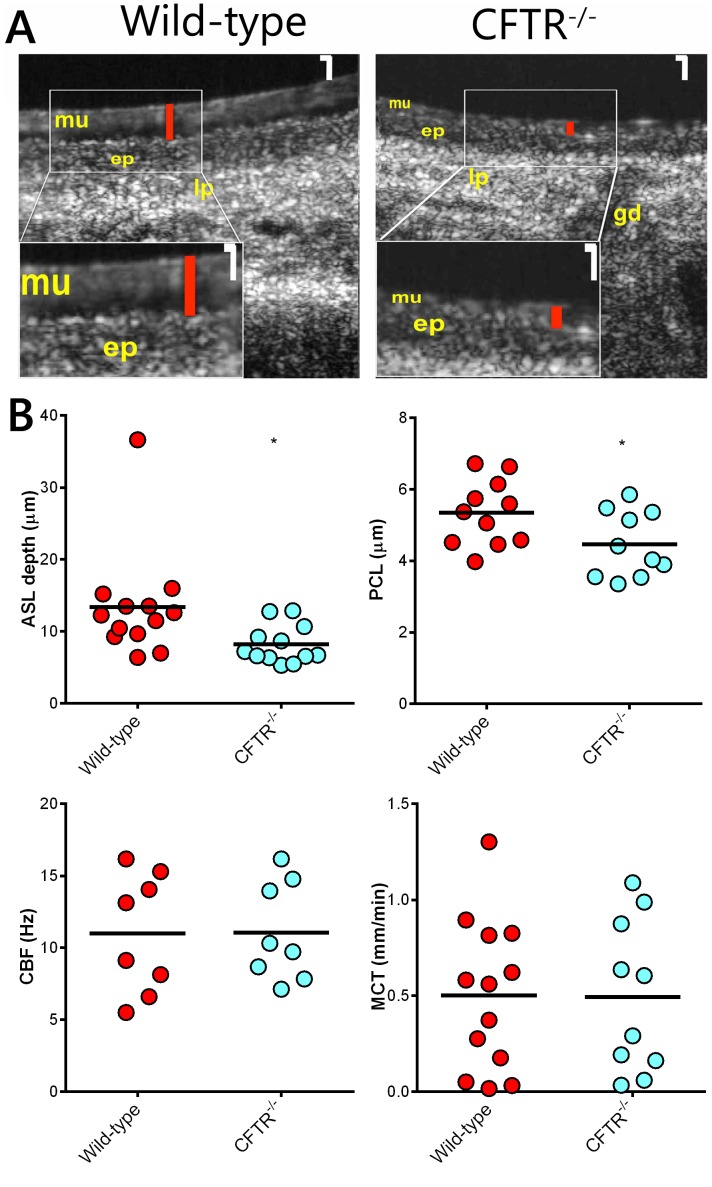
Functional anatomy of rat trachea. (A) Representative time-averaged µOCT images of wild-type and CFTR^−/−^ tracheas. Higher magnification insets (bottom left corner) show differences in ASL height between wild-type and CFTR^−/−^ animals. White magnification bar = 10 µm. Red bars indicate ASL height. Mucus layer (mu), epithelium (ep), lamina propria (lp), and gland duct (gd) are also visualized. (B) Summary data for airway surface liquid depth (ASL), periciliary liquid depth (PCL), ciliary beat frequency (CBF), and mucociliary transport (MCT). (n = 8–13 animals/group) *p≤0.05.

We used µOCT to visualize and quantify the functional microanatomy of the airway surface (Figure 6). Excised tracheas from wild-type animals had significantly greater ASL depth compared to CFTR^−/−^ rats (p≤0.05; Figure 6). In addition, PCL thickness was reduced in CFTR^−/−^ animals (p≤0.05). Ciliary beat frequency and mucociliary transport were not different between wild-type and CFTR^−/−^ groups.

### CFTR^−/−^ Male Rats Exhibit Bilateral Absence of the Vas Deferens

**Figure 7:**
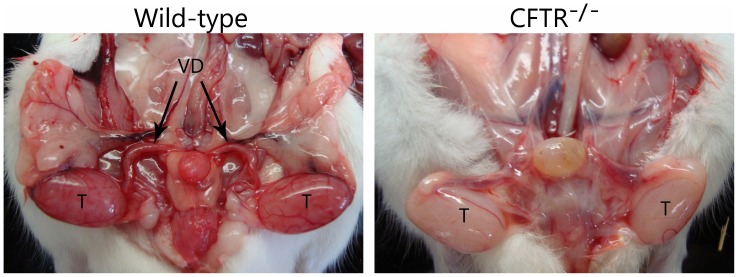
Male CFTR^−/−^ rats have bilateral absence of the vas deferens by 6 weeks of age. Wild-type males (left) have an intact reproductive tract. CFTR^−/−^ males (right) develop other reproductive organs, but exhibit absent vas at 6 weeks. T, testis; VD, vas deferens. (n = 3 animals/group).

Evaluation of gross genitourinary anatomy revealed absence of the vas deferens in 6 week old CFTR^−/−^ male rats (Figure 7). When sections from 6 month old males were evaluated histologically, only connective tissue and vasculature were observed with no evidence of the vas identified (data not shown).

### CF rats have Abnormal Dentition

**Figure 8:**
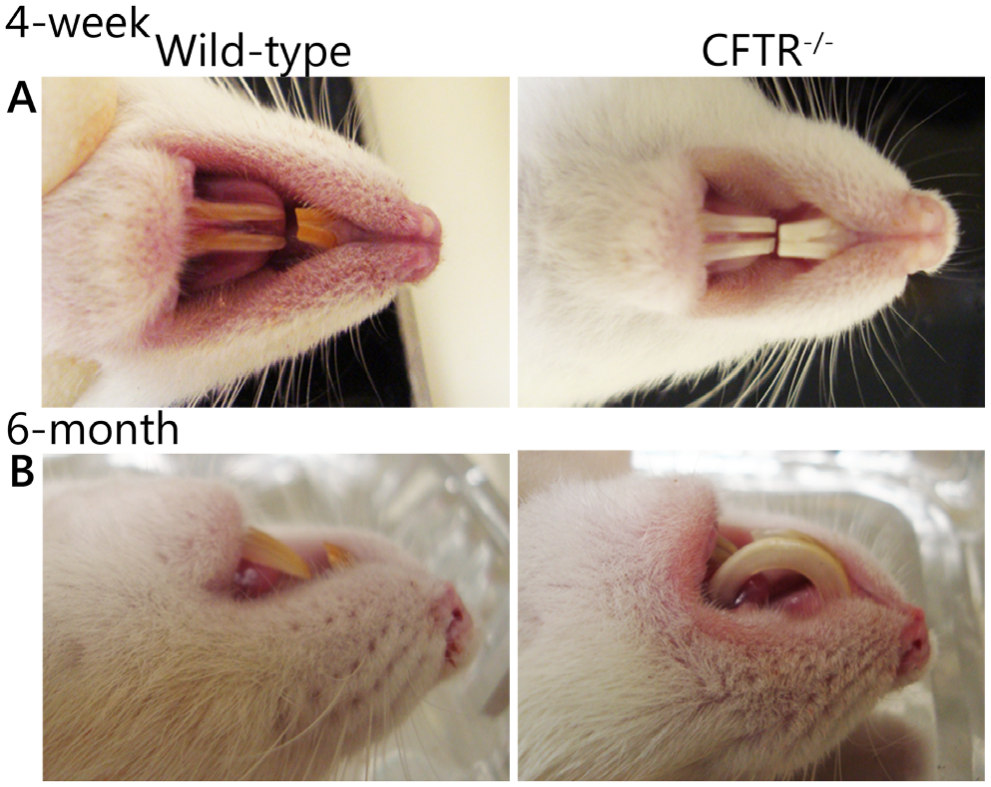
CF rats have abnormal dentition. (A) Wild-type rats (left) have yellowish-brown enamel while CFTR^−/−^ rats (right) exhibit bright white incisors. (B) Incisors from CF rats exhibit uncontrolled growth and penetrate the hard palate. (n = 3 animals/group).

Incisors of wild-type rats presented with yellow-brown enamel while CFTR^−/−^ rats exhibited white incisors (Figure 8A). Wild-type rats maintained normally filed teeth while CF animals exhibited grossly malformed dentition, possibly due to defective physiological trimming. Without intervention, incisors of CF rats developed dental malformations, including curvature of the incisors and penetration of the hard palate (Figure 8B).

### Lung and Major Exocrine Tissues from CFTR^−/−^ Rats are without Pathology or Inflammatory Cell Infiltrates During Early Life

Pulmonary sections from wild-type and CFTR^−/−^ rats appear to develop normally from days 22 to 42 (Figure S1). In addition, BAL profiles were not different between genotypic groups, indicating the absence of overt pulmonary inflammation. Pancreas and liver from young animals were also histologically normal (Figure S2).

### Hematology and Serum Chemistry

Hypoalbuminemia, decreased total protein, and increased blood urea nitrogen (Table S1) were observed in CFTR^−/−^ rats. Because many animals experienced substantial weight loss prior to euthanasia, these findings may be attributable in part to malnourishment and poor hydration. Serum electrolytes (Ca^2+^, Na^+^, K^+^) were not different between wild-type and CFTR^−/−^ groups (Table S1). A trend towards increased blood glucose levels was observed in CF animals with very high values in a subset (data not shown) which is currently under investigation.

Red blood cells, hemoglobin, and hematocrit were all within normal range for young animals. Total white blood cell counts were not different between groups, although the differential was influenced by genotype (Wild-type: 21±4% neutrophils and 76±4% circulating lymphocytes, CFTR^−/−^ animals: 52±5% neutrophils and 44±6% circulating lymphocytes), likely attributable to gastrointestinal or other stress-related complications observed in CFTR^−/−^ animals.

## Discussion

We present here a novel cystic fibrosis rat with a number of phenotypic characteristics that resemble the human disease. The CF rat furnishes a new opportunity to investigate (i) developmental processes that affect disease progression and severity, (ii) the impact of CFTR and other ion channels with regard to ASL depth in CF airways, (iii) contributions of ASL and PCL abnormalities to manifestations of CF respiratory pathology, and (iv) the role of CFTR in mucus synthesis and release.

Developmental defects attributable to absent CFTR have received considerable recent attention, including abnormal airway remodeling and tracheal formation [20], deficient levels of growth hormone [28], absent vas deferens [7], [29], and problems with enamel deposition in teeth [30]–[33]. Similar to CF pig [20] and human subjects [20], [34], we observed reduced tracheal circumference and a loss of submucosal gland area in the CF rat model (Figure 2), supporting the notion that CFTR may play a critical role during normal lung development [20]. The CF rat offers an ideal opportunity to monitor phenotypes such as these more longitudinally than has been practical in the past (e.g. from intrauterine tissue formation to adulthood).

The rat model will also serve as a valuable resource for investigating developmental events that lead to abnormalities of the male reproductive tract. The majority (∼99%) of men with CF are sterile due to degeneration or complete absence of the vas deferens [35]. In both the CF pig and ferret, the vas deferens is poorly formed or absent at birth [7], [29]. The most commonly utilized CF animal model, the mouse, does not exhibit this feature. CF rats have lost the vas deferens by 6 weeks of age. The role of chloride secretion versus intracellular or other CFTR functions with regard to vas deferens involution are not well understood, but can be more readily characterized *in vitro* and *in vivo* using this new animal model. The rat may also facilitate experiments intended to determine the extent to which vas tissue destruction can be ameliorated by early (e.g. prenatal) therapeutic intervention.

Another area of increasing interest is the development of dental complications in CF subjects. Similar to observations in mice [33], CFTR^−/−^ rats exhibit white incisors compared to yellow dentition of wild-type littermates (Figure 8A). Moreover, by three months of age CF rats demonstrate grossly malformed upper and lower incisors, characterized by curved dentition and penetration of the hard palate (Figure 8B). Additional studies are in progress to determine whether this phenotype is the result of (i) dysregulated growth and/or (ii) reduced wearing of the teeth, and the extent to which hypomineralization or other defects in enamel contribute to the findings [30], [31], [33], [36]. Human data indicate that many CF patients have diminished mineralization of enamel [37]–[40] which may predispose to development of caries. The rat represents an ideal means of further investigating this feature of clinical CF.

The relationship of ASL depth to pathogenesis of cystic fibrosis lung disease has engendered considerable controversy. Previous studies have concluded that depleted ASL is primarily responsible for cystic fibrosis pulmonary damage, and associated with defects in ciliary extension [41]. The present results in CFTR^−/−^ animals indicate ASL depletion in lower airways (Figure 6) that is qualitatively and quantitatively very similar to that noted in human subjects [42], but without evidence of overt respiratory pathology (Figure S1). Our findings establish that defective ASL hydration can occur for several weeks without resulting in manifestations of a CF pulmonary phenotype. While spontaneous lung disease is absent in very young CF rats, it is important to note that the animals described here have been housed in HEPA filtered ventilated cages. Future studies will determine whether older animals maintained under more natural housing conditions develop evidence of lower respiratory involvement. If such experiments fail to elicit a CF phenotype, the findings would indicate that defects in ASL and PCL hydration by themselves do not represent a proximate cause of CF pathology. The rat model will therefore furnish a valuable means to address unanswered questions regarding airway surface hydration and cystic fibrosis pathogenesis.

Decreased ASL depth has classically been attributed to Na^+^ hyperabsorption as a consequence of absent CFTR [43]–[46]. Similar to what has recently been reported in CF pig [47] and ferret [48], the cystic fibrosis rat does not exhibit sodium hyperabsorption in either nasal or lower airways. Although amiloride-sensitive sodium transport is not significantly different between wild-type and CFTR^−/−^ rats, ASL depth is markedly diminished (Figures 4 and 5). The abrogation of constitutively active CFTR fluid secretion may provide part of the explanation for ASL depletion in CF animals (as opposed normal levels of ASL observed in the CF porcine model) [47]. Our findings therefore suggest that CFTR (rather than ENaC) serves as the dominant regulator of ASL depth in the rat airway *in vivo*.

It has recently been argued that mucus in cystic fibrosis lungs is excessively viscous due to loss of CFTR dependent bicarbonate secretion, and that the newly formed mucus gel fails to properly expand [49]. We observed markedly increased mucus stores (i.e. cells appear ‘stuffed’ with mucins; Figure 4) in epithelial cells from the proximal nasal septa of CF compared to non-CF rats. Histological findings such as these are compatible with an intrinsic defect in mucus secretion/release [50]. CF pigs develop mucous cell hyperplasia [51] qualitatively similar to that observed in our young CFTR^−/−^ animals, and CF mice have also been reported to exhibit a phenotype characterized by measurably increased numbers of mucous cells [52]. Based on findings shown in Figure 4 and Table 1, the rat model should be useful for investigating CFTR-dependent airway mucin synthesis, expansion, and release.

The generation of the first CFTR^−/−^ rat using zinc finger nuclease technology suggests a clear path to other CF animal models with specific mutations relevant to therapeutic development, including animals that express F508del or CFTR premature truncations alleles. The advantages of a rat model for CF drug development include robust and multi-organ bioelectric and histological findings, ease of breeding, short gestation, manageable expense, availability of antibodies and other reagents for mechanistic studies, and standard use for pharmacokinetic and drug toxicity analysis.

In summary, the CFTR^−/−^ rat exhibits many features of the CF phenotype found in human subjects and CF animal models (summarized in Table 2), including defects in airway mucus production (Figure 4), tracheal development (Table 1), airway surface and periciliary fluid depth (Figure 6), nasal mucus (Figure 4), dentition (Figure 8), and involution of the vas deferens (Figure 7). Bioelectric findings in rat are also informative and demonstrate significantly diminished periciliary fluid without evidence of ENaC hyperactivity. Moreover, unlike the CF ferret or pig, the CFTR^−/−^ rat does not present with meconium ileus at birth. Only after weaning do large numbers of animals develop intestinal blockage, which appears similar to the distal intestinal obstruction syndrome observed in children and adults with cystic fibrosis [53]. Complications due to intestinal obstruction can be managed by use of dietary modification (Figure 2). The new CF rat model will provide a very useful resource for longitudinal studies of tissue development, electrophysiology, and other endpoints relevant to disease mechanism in the future.

**Table 2:** Summary of phenotypes across numerous CF species.

	Human	Mouse	Pig	Ferret	Rat
Increased Stored NasalMucus	NR	Yes [52]	Yes [51]	NR	Yes
Airway Surface Hydration	NR	Depleted [55]	Not Depleted [47]	NR	Depleted
Sodium Hyperabsorption	Yes [41], [56], No [57]	Yes [58], No [59]	No [47]	No [48]	No
Hypoplastic Tracheal Submucosal Glands	Inconclusive [20], [34]	NR	Hypoplastic at birth [20]	NR	Hypoplastic 21–42 DPN
Intestinal Obstruction	13–17% MI, 7–8% DIOS in childhood [60]	0–95% OB at weaning [4] a	100% MI [61]	50–100% MI [7]	∼70% OB between weaning and 42 DPN
Vas deferens	∼99% CBAVD [35]	Normal	Degenerate or absentvas at birth [29]	Degenerate or absentvas at birth [7]	Vas absent before 42 DPN
Dentition	Abnormal [37]–[39]	Abnormal [33]	Abnormal [31]	NR	Abnormal

CBAVD – congenital bilateral absence of the vas deferens, DIOS – distal intestinal obstruction syndrome, DPN – Days postnatal, NR – Not reported, MI – meconium ileus, OB – Obstruction.

a Background strain and/or CFTR genotype dependent.

## Supporting Information

Figure S1 **Analysis of lung tissue from CF rats.** (A) Lung histology of wild-type and CFTR^−/−^ animals. Magnification bar = 200 µm (n = 7–11 animals/group) (B) Total cell counts and cell differential of BAL in wild-type and CF rats (n = 4 animals/group).
(TIF)

Figure S2 **Histology of pancreas and liver from CF rats.** H&E stained paraffin sections of (A) pancreas and (B) liver from 22–44 day old wild-type and CFTR^−/−^ rats. Magnification bar = 100 µm (n = 3–5 animals/group).
(TIF)

Figure S3 **Expanded western blot (see** **Figure 2** **).** Western indicating absence of CFTR protein from lungs of CFTR^−/−^ animals and expression of CFTR in wild-type samples. Arrow - rat CFTR (∼150 kD as previously reported [54]); *indicates likely CFTR degradation product commonly observed in CFTR preparations. This experiment has been repeated three times in separate animals with similar results.
(TIF)

Table S1 **CBC and serum chemistry for wild-type and CFTR−/− rats.**
(DOCX)
