# Supplementary figures and images for: Characterization of Defects in Ion Transport and Tissue Development in Cystic Fibrosis Transmembrane Conductance Regulator (CFTR)-Knockout Rats
Source: PLoS One. 2014 Mar 7;9(3):e91253. doi: 10.1371/journal.pone.0091253 (PMC3946746; doi:10.1371/journal.pone.0091253)

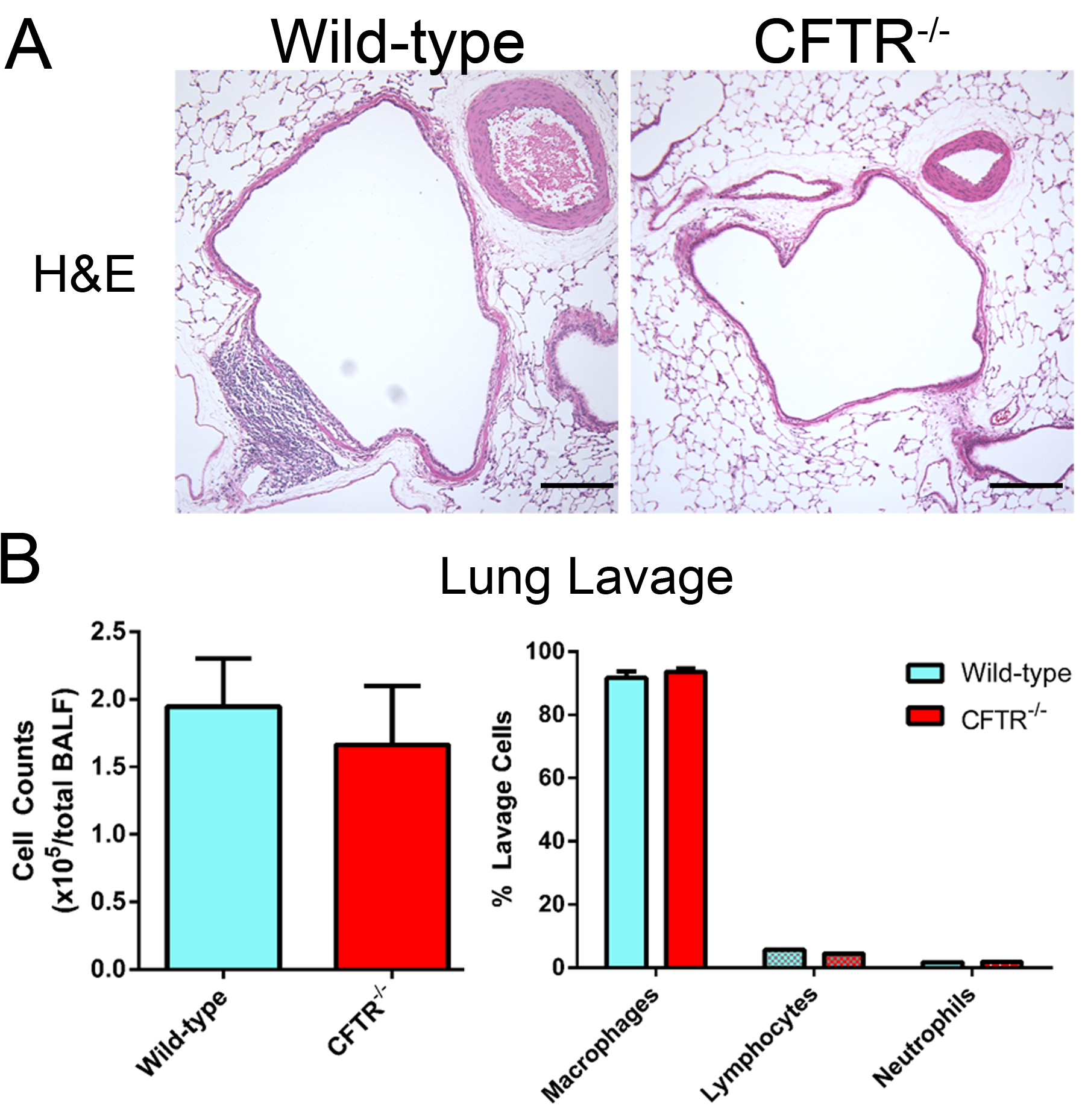

Supplement: Figure S1 — Analysis of lung tissue from CF rats. (A) Lung histology of wild-type and CFTR−/− animals. Magnification bar = 200 µm (n = 7–11 animals/group) (B) Total cell counts and cell differential of BAL in wild-type and CF rats (n = 4 animals/group). (TIF) [file pone.0091253.s001.tif]

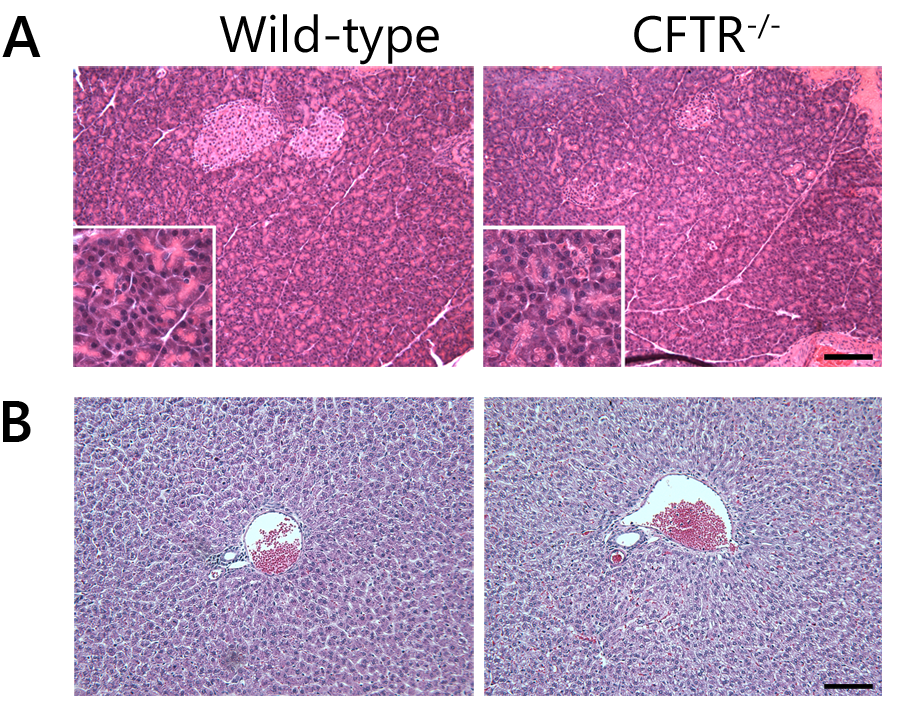

Supplement: Figure S2 — Histology of pancreas and liver from CF rats. H&E stained paraffin sections of (A) pancreas and (B) liver from 22–44 day old wild-type and CFTR−/− rats. Magnification bar = 100 µm (n = 3–5 animals/group). (TIF) [file pone.0091253.s002.tif]

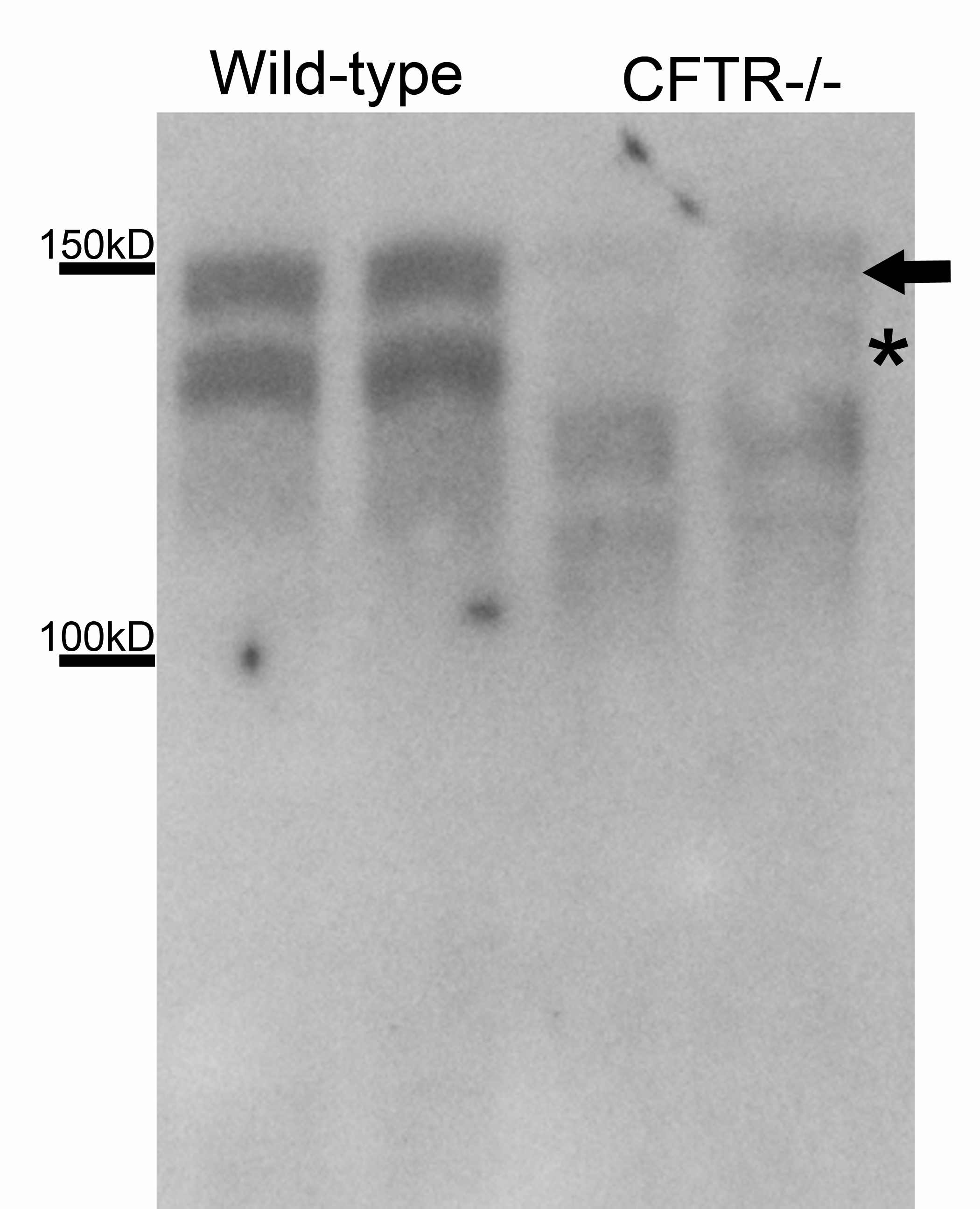

Supplement: Figure S3 — Expanded western blot (see Figure 2 ). Western indicating absence of CFTR protein from lungs of CFTR−/− animals and expression of CFTR in wild-type samples. Arrow - rat CFTR (∼150 kD as previously reported [54]); *indicates likely CFTR degradation product commonly observed in CFTR preparations. This experiment has been repeated three times in separate animals with similar results. (TIF) [file pone.0091253.s003.tif]
