# Supplementary material for: Characterization of Defects in Ion Transport and Tissue Development in Cystic Fibrosis Transmembrane Conductance Regulator (CFTR)-Knockout Rats
Source: PLoS One. 2014 Mar 7;9(3):e91253. doi: 10.1371/journal.pone.0091253 (PMC3946746; doi:10.1371/journal.pone.0091253)
Supplement: Table S1 — CBC and serum chemistry for wild-type and CFTR−/− rats. (DOCX) [file pone.0091253.s004.docx]

**Table S1.** **CBC and serum chemistry for wild-type and CFTR^-/-^ rats.**

|  | Wild-type | CFTR^-/-^ |
| --- | --- | --- |
| ***Complete Blood Counts*** |  |  |
| WBC (10^3^/µL) | 4.2 ± 0.6 | 4.0 ± 0.4 |
| Neutrophils | 793 ± 119 (21%) | 2053 ± 220 (52%)** |
| T Lymphocytes | 3280 ± 575 (76%) | 1804 ± 322 (44%)* |
| RBC (x10^6^/µL) | 5.3 ± 0.1 | 5.6 ± 0.3 |
| Hemoglobin (g/dL) | 12.1 ± 0.3 | 11.9 ± 0.6 |
| Hematocrit (%) | 35.9 ± 1.1 | 37.3 ± 1.9 |
| ***Serum Chemistry*** |  |  |
| Albumin (g/dL) | 4.1 ± 0.1 | 3.2 ± 0.2** |
| Alkaline Phosphatase (U/L) | 488 ± 67 | 173 ± 20* |
| ALT (U/L) | 56 ± 4 | 40 ± 4* |
| Amylase (U/L) | 756 ± 23 | 532 ± 40* |
| BUN (mg/dl) | 12.1 ± 0.7 | 17.8 ± 1.4* |
| Total Bilirubin (mg/dL) | 0.23 ± 0.01 | 0.23 ± 0.01 |
| Glucose (mg/dL) | 160 ± 9 | 224 ± 37 |
| Total Protein (g/dL) | 4.7 ± 0.1 | 4.2 ± 0.2* |
| Globulin (g/dL) | 0.69 ± 0.05 | 0.96 ± 0.04 |
| Ca^++^ (mg/dL) | 10.7 ± 0.2 | 11.0 ± 0.4 |
| Phosphate (mg/dL) | 11.8 ± 0.6 | 12.1 ± 0.7 |
| Na^+^ (mmol/L) | 140 ± 1 | 141 ± 1 |
| K^+^ (mmol/L) | 6.3 ± 0.4 | 7.6 ± 0.5 |

Values shown as mean ± SEM (n=7 animals/group for CBC, n=14-15 animals/group for serum chemistry)

*p ≤ 0.05, **p ≤ 0.001 (Student’s t-test)
